# Draft Genome Sequence of a Shewanella halifaxensis Strain Isolated from the Intestine of Marine Red Seabream (Pagrus major), Which Includes an Integrative Conjugative Element with Macrolide Resistance Genes

**DOI:** 10.1128/genomeA.00297-18

**Published:** 2018-04-19

**Authors:** Yuta Sugimoto, Fumito Maruyama, Satoru Suzuki

**Affiliations:** a Center for Marine Environmental Studies, Ehime University, Matsuyama, Japan; b Graduate School of Medicine, Kyoto University, Kyoto, Japan

## Abstract

Shewanella halifaxensis strain 6JANF4-E-4 was isolated from the intestine of a red seabream (Pagrus major). Here, we report the draft genome sequence of this bacterium, which includes an integrative conjugative element of the SXT/R391 family, where the macrolide resistance determinants *mef*(C) and *mph*(G) exist.

## GENOME ANNOUNCEMENT

The genus Shewanella contains facultative anaerobic Gram-negative rod bacteria, which are often found in marine environments. Shewanella halifaxensis was recorded from marine sediment (1). Our strain, S. halifaxensis 6JANF4-E-4, was isolated from the intestine of a red seabream (Pagrus major), and it shows macrolide resistance and possesses the tandem gene set of *mef*(C)-*mph*(G) (2). We revealed the alignment of *mef*(C) and *mph*(G) on a chromosome and their integration within an integrative conjugative element of the SXT/R391 family (SRI).

We provide here the draft genome sequence of a gene cassette containing *mef*(C)-*mph*(G) in the SRI. SRIs were first described in Vibrio cholerae strains isolated from human clinical specimens and have been found in several species of Vibrio, Photobacterium, Providencia, Proteus, Alteromonas, Marinomonas, and Shewanella (3).

The genome sequencing of the 6JANF4-E-4 strain was performed by shotgun sequencing using the PacBio RS II system (Pacific Biosciences). The 87,318 sequence reads were pooled and *de novo* assembled using single-molecule real-time (SMRT) Portal analysis software version 2.3 (Pacific Biosciences) (4). The draft genome of the strain was 5,462,838 bp, with an average G+C content of 42.8%, and consisted of 5 contigs (average contig length, 1,092,567 bp). Rapid Annotations using Subsystems Technology (RAST) and SEED Viewer were used for gene annotation and an overview of the annotated genome, respectively (5, 6), and revealed 5,068 coding sequences and 177 RNA genes on this genome.

An SRI has the specific sequences *attL* and *attR* (3) at opposite ends. We confirmed that *mef*(C)-*mph*(G) was coded between *attL* and *attR* on the SRI. Other antibiotic resistance genes, namely *floR*, *sul2*, and a putative β-lactamase gene, were also coded. We assigned the nomenclature ICE*Sha*Jpn1, according to the international naming standard for the SRI (7). The length of ICE*Sha*Jpn1 was 107,133 bp and contained the functional genes for maintenance, dissemination, and regulation as an SRI (8).

### Accession number(s).

The S. halifaxensis strain 6JANF4-E-4 genome sequence data have been deposited in DDBJ/EMBL/GenBank under accession numbers BFBQ01000001 to BFBQ01000005. The released sample is available on the DDBJ BioSample listing page under sample number SAMD00112641.
